# Supplementary material for: The impact of the National Programme to Eliminate Lymphatic Filariasis on filariasis morbidity in Sri Lanka: Comparison of current status with retrospective data following the elimination of lymphatic filariasis as a public health problem
Source: PLoS Negl Trop Dis. 2024 Aug 14;18(8):e0012343. doi: 10.1371/journal.pntd.0012343 (PMC11324254; doi:10.1371/journal.pntd.0012343)
Supplement: Attachment — Submitted filename: review report_PNTD-D-23-01643.docx [file pntd.0012343.s003.docx]

**Comment on the ms on “The impact of the National Programme to Eliminate Lymphatic Filariasis on filariasis morbidity in Sri Lanka: comparison of current status with retrospective data following the elimination of lymphatic filariasis as a public health problem PNTD-D-23-01643” by Gunarathne et al.**

This manuscript aims to assess the impact of five rounds of MDA with DA (2002-2006) on LF disease manifestations (lymphoedema and hydrocele) sixteen years after stopping MDA. The authors using data on passive case detection on lymphoedema (from filariasis clinics) and hospital admission on hydrocele (hospital records) explored the trends in (i) passive case detection (PCD) rates (ratio of the no. of cases detected to that of estimated population in 2022) for lymphoedema by district (2012-2022) and overall (from 2006 -2022), and (ii) hydrocele admission rates from 2007-2017 by district / province and overall. Globally, very few studies have examined the impact of MDA on LF chronic disease manifestations and the results are highly variable. As WHO is targeted to achieve LF elimination by 2030, the results of this study are important to know the status of global effort to eliminate LF as a public health problem.

The manuscript is well written with clear background and objectives. The results are presented well with figures and Tables, and the discussion is narrated comparing results available from studies elsewhere. While I recommend the ms for publication, I would request the authors to provide a detailed analysis of this historical data on clinical manifestations.

**Major comments**

While trend analyses show the impact of NPELF on disease manifestation (lymphoedema & hydrocele) over time, it would be interesting to examine the impact on preventing new cases in different age-classes, particularly in younger population (<20 yrs, it is <3% in 2022). In this respect, I would suggest graphical comparison of the PCD rates between 2012 and 2022 by age-class separately for each district, province and overall (Sri Lanka) and a statistical comparison of the rates between 2012 and 2022.

As majority (74%) of the diseased in 2022 are in grades 1 and 2 (early stage, Table 1), a comparison of age-distribution of early stage of lymphoedema between 2012 and 2022 for each district / province and overall, would help to assess the impact of NPELF on disease incidence following MDA. A similar analysis can also be carried out for late stages of lymphoedema, and hydrocele.

**Minor comments**

**Methods**

Trend analyses was carried out showing regressing lines relating PCD rates with time and R^2^ value. Since the rates are proportions, the normality assumption is not valid for this type of data. It is not clear whether the authors normalized the response variable (PCD rate) before doing least-square regression analysis. Alternatively, the degree and the direction of relationship can be examined using the Spearman Rank correlation coefficient. Also, please provide p-values for statistical significance.

**Results**

***LL 164-171***

Table 1: Please insert a column and provide population data for each age-class and calculate PCD rates per 10000 population for each age-class. Similarly, provide gender wise population total and PCD rates.

The lymphoedema PCD rates differ among districts. Whether the impact of NPELF on LF disease (also early stage of lymphoedema, i.e. grade 1 and 2) is associated with Mf-prevalence in the districts prior to or after stopping MDA.

**Discussion**

LL 212-213: Note that the frequency of cases was highest in Colombo (108) and Kalutara (113) (not in Gampaha, the cases are only 52) (Table 1). Please make appropriate correction and revise the text.

LL213-214: Please verify whether the case distribution is per population or per 10000 population. Table 1, did not provide data on PCD rates to make this comparison. Please revise Table 1 as per comments in the Results section and revise the text accordingly.

LL228-230: What for conservative estimate of achieving a 14% reduction in lymphoedema prevalence was assumed. Please make it clear, else this sentence may be deleted as it has no relevance in this context.
