# Supplementary material for: The impact of the National Programme to Eliminate Lymphatic Filariasis on filariasis morbidity in Sri Lanka: Comparison of current status with retrospective data following the elimination of lymphatic filariasis as a public health problem
Source: PLoS Negl Trop Dis. 2024 Aug 14;18(8):e0012343. doi: 10.1371/journal.pntd.0012343 (PMC11324254; doi:10.1371/journal.pntd.0012343)
Supplement: Attachment — Submitted filename: Rsponses to reveiwers comments.docx [file pntd.0012343.s004.docx]

Responses to Reviewer's Queries

<b>Key Review Criteria Required for Acceptance?</b></br></br>
As you describe the new analyses required for acceptance, please consider the following:</br></br>

<b>Methods</b></br></br>
-Are the objectives of the study clearly articulated with a clear testable hypothesis stated?</br>
-Is the study design appropriate to address the stated objectives?</br>
-Is the population clearly described and appropriate for the hypothesis being tested?</br>
-Is the sample size sufficient to ensure adequate power to address the hypothesis being tested?</br>
-Were correct statistical analysis used to support conclusions?</br>
-Are there concerns about ethical or regulatory requirements being met?</br></br>

Reviewer #1: Yes to all, but suggestion for a detailed analysis is given in the attached review report.

Reviewer #2: **Comment; Objective is relatively clear however the methodology needs improving.
The study design is not clear. It does not provide rational for the different years of hydrocoele and lymphoedema cases collected**.

**Response;** The study design was a retrospective analysis of the passive case detection data to see the trends in case detection of lymphoedema and hydrocele over time. In the revised manuscript we have retrieved the data for missing years (2018- 2022) by a special request to the Medical Statistics Division, Ministry of Health, thus the period of analysis is comparable (up to 2022 for both lymphoedema and hydrocele).

**Comment; Population estimates are based on the 2012 census. It does not seem to have taken population growth into account over the long time period. Adjustments need to be considered**.

**Response;** The estimated population for the relevant year based on 2012 census was used in the calculation. This annual population estimate takes into account population growth (births, deaths, migration etc.)

**Comment; More clarity on the hydrocoele admission process, it is free for all patients, do the have access, waiting lists. Why were hospital data not included to 2022?**

**Response;** In Sri Lanka patients have the freedom to select any hospital in any region to obtain medical services. Medical services are free of charge in the state medical sector but there are waiting lists for routine or planned medical procedures. Hospital admission data of all state hospitals in the country are compiled in the indoor morbidity and mortality register under the Division of Medical statistics of the Ministry of Health. The hydrocele data used in the initial analysis (2007-2017) was available at the Anti- Filariasis campaign derived from this central source in 2018. In the revised manuscript the data till 2022 has been included by obtaining the data by a special request .

**Comment; No mention of how reporting systems may have changed over time - this could influence the results**.

**Response;** There were a few changes in the reporting system that has been stated under Methods, lines 165-166. District wise reporting of data for lymphoedema was available from 2012, demographic data , Age classification from 2019 and grading of lymphoedema from 2020.

**Comment; No mention of ethics approval for accessing clinic or hospital records**.

**Response;** This has been included under Methods, lines 150-152. **T**he project was an audit of existing and archived data maintained by the National Anti-Filariasis Campaign (AFC). The study was eligible for ethics exemption for the following reasons; the study was based on analysis of archived data maintained at AFC and the current data used was in a public domain. The study did not at any stage involve collection or use of individual level or community level data on sensitive topics. No biological samples were involved.

Reviewer #3: - Objectives stated clearly
**Comment;- If you have data on population and prevalence, please consider to add DALYs.**

**Response;** Since we do not have morbidity prevalence (it was passive case detection rates of new lymphedema cases) DALYs cannot be calculated**.**

.
**Comment;**- **Unclear how many hospitals/where and if representative sample for the whole of Sri Lanka. So unclear if population is appropriate**.

**Response;** All state hospital data were included for inpatient management of hydroceles.

**- Comment; Are there no updated census data available? This has implications for the results, need to discuss in the Discussion**

**Response;** The estimated population for the relevant year was utilized (this data is updated based on the annual population growth rate (include birth, deaths, migration etc.). See under Methods, lines 157-158 and163-164.

**Comment**;- **How to ensure anonymity of patient records collected for the study?**

**Response**; Individual level data were not utilized, archived data were used in the retrospective analysis and the 2022 data was derived from a public domain.

-**Comment;** **Why hydrocele data only till 2017? This is a great limitations of this study. Are the patient database comparable between lymphoedema and hydrocele?**

**Response;** This has been attended. ( The data from 2018-2022 was obtained from the Medical statistics Division of the Ministry of Health by a special request. --------------------

<b>Results</b></br></br>
-Does the analysis presented match the analysis plan?</br>
-Are the results clearly and completely presented?</br>
-Are the figures (Tables, Images) of sufficient quality for clarity?</br></br>

Reviewer #1: (1) Yes but needs clarity on the methods used for statistical Analysis.
(2) Results are clear and presented well, more results based on detailed analysis is suggested.

**Response;** The results were re-analyzed. Poisson log-linear models were used to assess the trends in PCD for endemic regions, including district-wise trends and hospital admissions for the management of hydroceles/spermatoceles. see lines 168-174 and 177-183 in the revised manuscript.

(3) Figures are with good quality and useful for interpretation.

Also, please see the attached review report for a detailed analysis and presentation of results.

Reviewer #2: **Comment**; **The case numbers are presented for 2022 – are there similar numbers and demographic information for the other years? The difference in average age in 2006 to 2022 would be informative**.

**Response;** Demographic information for lymphoedema was documented from 2019 onwards. The lymphoedema case numbers by age categories have been included in a new Table (Table 2) in the revised manuscript. The average age is similar for the said years.

**Comment; The figures' resolution could be improved . All data in the figures and tables need to be included as supplementary files.**

Response; All data are in supplementary files. A new set of figure are included to the revised manuscript.

Reviewer #3: - **Comment;** **How come that there are so many new cases of morbidity among young people (<21 years)? Does this mean that there is still circulating infection?**

**Response**; Lymphoedema cases among less than 20 years was 3% (n=15) in 2022. A new paragraph summarizing the possibilities for occurrence of lymphoedema has been included to the revised paper under Discussion. Lines 333-339.

**Comment; - Please add a geographic map with per region the levels/categories of clinical morbidity over time periods, such as to see straight in which districts potential transmission still occurs, and where more MMDP needs to be invested.**

**Response;** This has been attended see figure 1.  **Comment;- Provide (%) in Table 1, to compare variables**

**Response**; This has been included, thank you for pointing it out.  **Comment; - Actually only Colombo (most cases), Kurunegala, and Gampaha are declining in cases. This is an interesting result, and it needs to be described more clearly why no decline is seen in the other areas. What challenges contribute to a lack in decline?**

**Response**; The analysis show that there was a significant decline in the overall PCD rates for lymphoedema specifically in the 3 districts, Colombo, Gampaha and Kurunegala a steady decline was noted. Except for Kalutara (static PCD rate) the other districts also show a recent declining trend. The possible reasons for the variable declines in the morbidity rates have been discussed lines-324-332. **Comment;- Sri Lanka overall results are mostly driven by Colombo, and this needs to be stated somewhere. There actually is no country-wide decline.**

**Response;** The overall decline in PCD rates was significant. According to the detailed analysis included in the revised manuscript except for the district of Kalutara (static trend) all other districts show a recent declining trend for lymphoedema PCD rates

**. Comment;- Are there no regional figures to be made for hydrocele?**

**Response;** We did not focus on regional analysis for hydrocele management as people have the freedom to seek surgical or medical attention from any hospital of their choice ,thus to avoid waiting lists they may present to a hospital outside the endemic region. Also to capture migrants from endemic regions (employment, marriage etc)

<b>Conclusions</b></br></br>
-Are the conclusions supported by the data presented?</br>
-Are the limitations of analysis clearly described?</br>
-Do the authors discuss how these data can be helpful to advance our understanding of the topic under study?</br>
-Is public health relevance addressed?</br></br>

Reviewer #1: Yes to all

Reviewer #2**: Comment; The discussion needs to highlight more on why there are differences in the rates of decline or why there is no decline in the number of cases - i..e what are potential reasons - socio-economic improvement in Colombo may have important, or in another areas the evidence of ongoing transmission may have helped to maintain case numbers.
Overall referencing needs to be improved. Seems more focus on transmission than morbidity**.

**Response;**. A new paragraph has been included under Discussion, lines 324-332 highlighting the reasons for variable declines in PCD rates of lymphoedema across districts.

**Comment; Seems more focus on transmission than morbidity**

**Response;** Focusing on transmission was unavoidable when discussing reasons for variability in decline of disease rates.

Reviewer #3: **- Comment**; **Very long discussion. Needs to be more comprehensive, and to the point.
Response;** The discussion has been shortened where possible.

**Comment;- If Tx was provided, you expect a halt in progression of tissue damage. Thus a halt in development of clinical disease. Still, in this study, the authors report new clinical disease, primarily among the young population. I think this data must be coupled with (historical) MF prevalence data to get a better understanding of why we see these new lymphoedema cases arising.**

**Response;** The question is not very clear. It is a known fact that filarial lymphoedema takes a fairly long time to manifest (even a decade) following infection (transmission event) and once disease is established tissue damage will progress even in the absence of the parasite (disease outlives the parasite). In Sri Lanka there is on-going low-level transmission in high endemic pockets. A new paragraph has been included under Discussion in the revised manuscript detailing the possibilities for occurrence of new lymphoedema cases lines 333-339.

**Comment;- Very long description of what is found in other countries (lines 215-232), but we need to get a grip of what happens in Sri Lanka, which is missing here.**

**Response;** The authors have omitted some of the descriptions of MDA outcomes in other countries to make the discussion shorter. The current LF situation in Sri Lanka has been briefly discussed ( two paragraphs) lines 308-323.

**Comment; Lines 236-239, infection parameters are mentioned, but not shown, whereas this data is critical to understand the results of this study**

**Response ;**The reference for infection parameters mentioned in the discussion is included to the revised manuscript.

--------------------

<b>Editorial and Data Presentation Modifications?</b></br><br/>
Use  this section for editorial suggestions as well as relatively minor modifications of existing data that would enhance clarity. If the only modifications needed are minor and/or editorial, you may wish to recommend “Minor Revision” or “Accept”.

Reviewer #1: (No Response)

Reviewer #2: (No Response)

Reviewer #3: **Comment; Overall prospects of an interesting paper, and great to see the progress Sri Lanka is making**.

**Response** Thank You
- Create some space between x-axis and y-axis and legends in the figures.
- Overall pixels are very low of the figures. Please submit again with improved pixel-level.
- R-squared value is often overwriting the histograms of the figures

Response; A new set of figures have been included with reanalysis of data where these deficiencies are not issues.
--------------------

<b>Summary and General Comments</b></br></br>
Use  this section to provide overall comments, discuss strengths/weaknesses of the study, novelty, significance, general execution and scholarship. You may also include additional comments for the author, including concerns about dual publication, research ethics, or publication ethics. If requesting major revision, please articulate the new experiments that are needed.

Reviewer #1: This manuscript aims to assess the impact of five rounds of MDA with DA (2002-2006) on LF disease manifestations (lymphoedema and hydrocele) sixteen years after stopping MDA. The authors using data on passive case detection on lymphoedema (from filariasis clinics) and hospital admission on hydrocele (hospital records) explored the trends in (i) passive case detection (PCD) rates (ratio of the no. of cases detected to that of estimated population in 2022) for lymphoedema by district (2012-2022) and overall (from 2006 -2022), and (ii) hydrocele admission rates from 2007-2017 by district / province and overall. Globally, very few studies have examined the impact of MDA on LF chronic disease manifestations and the results are highly variable. As WHO is targeted to achieve LF elimination by 2030, the results of this study are important to know the status of global effort to eliminate LF as a public health problem.

The manuscript is well written with clear background and objectives. The results are presented well with figures and Tables, and the discussion is narrated comparing results available from studies elsewhere. While I recommend the ms for publication, I would request the authors to revise ms with results based on a detailed analysis of this historical data on clinical manifestations, before accepting for publication.

Response; The data has been re-analyzed in the revised manuscript with inclusion of maps to show the declining rates with time in the endemic region. Poisson log-linear models were used to assess the trends in PCD for endemic regions, including district-wise trends and hospital admissions for the management of hydroceles/spermatoceles. Age wise categorization of lymphedema has been included from 2019 onwards for each endemic district (this is the available data).

**Comment on the ms on “The impact of the National Programme to Eliminate**

**Major comments**

**Comment; While trend analyses show the impact of NPELF on disease manifestation (lymphoedema & hydrocele) over time, it would be interesting to examine the impact on preventing new cases in different age-classes, particularly in younger population (<20 yrs, it is <3% in 2022). In this respect, I would suggest graphical comparison of the PCD rates between 2012 and 2022 by age-class separately for each district, province and overall (Sri Lanka) and a statistical comparison of the rates between 2012 and 2022**.

**Response**; PCD rates by age class cannot be calculated due to lack of population data. Only the overall estimated population data is available to use as the denominator

**Comment; As majority (74%) of the diseased in 2022 are in grades 1 and 2 (early stage, Table 1), a comparison of age-distribution of early stage of lymphoedema between 2012 and 2022 for each district / province and overall, would help to assess the impact of NPELF on disease incidence following MDA. A similar analysis can also be carried out for late stages of lymphoedema, and hydrocele.**

**Response** ;Age distribution has been documented from 2019 and staging was introduced from 2020.

**Minor comments**

**Methods**

**Comment; Trend analyses was carried out showing regressing lines relating PCD rates with time and R^2^ value. Since the rates are proportions, the normality assumption is not valid for this type of data. It is not clear whether the authors normalized the response variable (PCD rate) before doing least-square regression analysis. Alternatively, the degree and the direction of relationship can be examined using the Spearman Rank correlation coefficient. Also, please provide p-values for statistical significance**.

The data was reanalyzed . Separate Poisson loglinear models were used to assess the overall and district-wise PCD rates over the years. R programming language 4.4.0 was used for analysis. See under Methods lines 168-183.

**Results**

**Comment; *LL 164-171***

**Table 1: Please insert a column and provide population data for each age-class and calculate PCD rates per 10000 population for each age-class. Similarly, provide gender wise population total and PCD rates.**

**Response**; Unfortunately this is not possible as yearly population estimates are not available for separate age classes.

**Comment; The lymphoedema PCD rates differ among districts. Whether the impact of NPELF on LF disease (also early stage of lymphoedema, i.e. grade 1 and 2) is associated with Mf-prevalence in the districts prior to or after stopping MDA.**

This has been dealt with in under Discussion, 308-323.The lymphoedema PCD rates were higher in the districts of Matara and Galle where MF rates were the highest prior to MDA (high baseline prevalence) and MF and infective rates remained high in these districts even after the cessation of MDA requiring additional rounds of mop MDAs in 2013, 2014. Similarly, in the district of Puttalam, and rates were high with emergence of zoonotic brugian filariasis reported during the post MDA phase.

**Discussion**

**Comment; LL 212-213: Note that the frequency of cases was highest in Colombo (108) and Kalutara (113) (not in Gampaha, the cases are only 52) (Table 1). Please make appropriate correction and revise the text.**

**Response;** Thank you. Corrected.

**Comment; LL213-214: Please verify whether the case distribution is per population or per 10000 population. Table 1, did not provide data on PCD rates to make this comparison. Please revise Table 1 as per comments in the Results section and revise the text accordingly.**

Case distribution rate was per 10 000 estimated population for the relevant year. Table 1 was revised as per comments.

**Comment; LL228-230: What for conservative estimate of achieving a 14% reduction in lymphoedema prevalence was assumed. Please make it clear, else this sentence may be deleted as it has no relevance in this context**.

Response; Thise statement was deleted as the assumption was not clear.

**Reviewer #2: The paper's aim of looking at changes in case numbers over time is an important one, however, the methods and results need to be improved. The paper seems a little superficial. Language and grammar needs improving. Some references quite old/outdated. e.g. ref #10 is old to refer to inconsistency in numbers or just lacking in relation to big statements e.g. line 290. Inconsistencies with abbreviation usage - LF vs. lymphatic filariasis**

**Response**; The Methods and results would have undoubtably improved with the reanalysis of data in the revised manuscript along with the overall quality of the paper. The paper was based on passive case detection data which is scientifically less robust than active case detection. This has been mentioned as a limitation.

**Comment “Some references quite old/outdated. e.g. ref #10 is old to refer to” Response;** Some of the references on pathology and morbidity outcomes following preventive chemotherapy were old. Outdated I do not agree as these references are still cited and valid.

**Comment “just lacking in relation to big statements e.g. line 290”**

**Response;** Sorry, **t**he aim of the manuscript was to disseminate the findings of the study clearly to the scientific community in simple language.

**Comment “Inconsistencies with abbreviation usage - LF vs. lymphatic filariasis”**

Have checked but failed to detect inconsistencies in abbreviation LF usage. Kindly point out where.

Introduction
-       **The clinical manifestations need to be described better.**

I agree. I have expanded the descriptions limiting to chronic manifestations since the focus of the paper was on chronic manifestations in the revised manuscript, under Introduction, lines 93-96.

-       **It would be useful to have a map of the endemic districts and provinces**

**Response; Attended**

**-       The overall rate of MF in 2001 is very low – what was the variability/range across the endemic districts at this time?**

**Response;** In 2001 the MF rate varied across endemic districts from 0.09- (Hambantota) to 0.41 (Galle)

-       **What does post–validation surveillance entail – a brief description would be helpful**.

Thank you. A brief description has been included under Introduction, see lines 123-126.

**-       The statement that LF morbidity has not been assessed doesn’t seem to take other pieces of work into account  - There are a few papers that refer to the burden and also morbidity e.g.**  [**https://pubmed.ncbi.nlm.nih.gov/18060080/**](https://pubmed.ncbi.nlm.nih.gov/18060080/)   [**https://pubmed.ncbi.nlm.nih.gov/16004709/**](https://pubmed.ncbi.nlm.nih.gov/16004709/)   [**https://pubmed.ncbi.nlm.nih.gov/29175490/**](https://pubmed.ncbi.nlm.nih.gov/29175490/) **The lack of references to other morbidity papers in Sri Lanka suggest the co-authors need to do more background reading.**

**Response;** These two references were included in the list of references (references 11, 12)and cited in the text in lines 119, 121. What was meant was that the nationwide burden of LF morbidity has never been assessed. Lines 126-127.

**Comment How has reporting systems changed / if they have changed**
**Response;** There were a few changes in the reporting system that has been stated under Methods, lines 165-166. District wise reporting of data for lymphoedema was available from 2012, age categorization in 2019 and grading of lymphoedema from 2020.

**Reviewer #3: - Interesting paper and nice that they looked at clinical morbidity. Overall prospects of an interesting paper, and great to see the progress Sri Lanka is making.
- Why mention R2 in abstract if not explained. Also no hydrocele rates mentioned in the abstract. Need to use same approach of reporting for lymphoedema and hydrocele.
- Hydrocele data and analyses are unfortunately very limited and difficult to compare with the lymphoedema data (other years, other hospitals?, no regional data, etc)
- Please add the database as a Supplement**

**Response;** Thank you for your words of encouragement. The data has been reanalyzed. Poisson log-linear models were used to assess the trends in PCD for endemic regions, including district-wise trends and hospital admissions for the management of hydroceles/spermatoceles. R programming language 4.4.0 was used in the analysis.

Database has been added as a supplement. We have rectified the limitations in hydrocele management data in the revised manuscript.

PLOS authors have the option to publish the peer review history of their article (<a href="<https://journals.plos.org/plosntds/s/editorial-and-peer-review-process#loc-peer-review-history>" target="_blank">what does this mean?</a>). If published, this will include your full peer review and any attached files.<br><br>

If you choose “no”, your identity will remain anonymous but your review may still be made public.<br><br>

<b>Do you want your identity to be public for this peer review?</b> For information about this choice, including consent withdrawal, please see our <a href="<https://www.plos.org/privacy-policy>" target="_blank">Privacy Policy</a>.

Reviewer #1: No

Reviewer #2: No

Reviewer #3: No


Figure Files:
While revising your submission, please upload your figure files to the Preflight Analysis and Conversion Engine (PACE) digital diagnostic tool, [https://pacev2.apexcovantage.com](https://pacev2.apexcovantage.com/). PACE helps ensure that figures meet PLOS requirements. To use PACE, you must first register as a user. Then, login and navigate to the UPLOAD tab, where you will find detailed instructions on how to use the tool. If you encounter any issues or have any questions when using PACE, please email us at [figures@plos.org](mailto:figures@plos.org).
